# Monitoring System of the Mar Menor Coastal Lagoon (Spain) and Its Watershed Basin Using the Integration of Massive Heterogeneous Data †

**DOI:** 10.3390/s22176507

**Published:** 2022-08-29

**Authors:** Francisco Javier López-Andreu, Juan Antonio López-Morales, Joaquín Francisco Atenza Juárez, Rosa Alcaraz, María Dolores Hernández, Manuel Erena, Jose Antonio Domínguez-Gómez, Sandra García Galiano

**Affiliations:** 1 Institute of Agricultural and Environment Research and Development of Murcia—IMIDA, Mayor Street, La Alberca, 30150 Murcia, Spain; 2 Department of Mining and Civil Engineering, Universidad Politécnica de Cartagena, 30203 Cartagena, Spain

**Keywords:** software applications, environment, land use, monitoring, heterogeneous data, big data, interoperability, machine learning

## Abstract

The tool created aims at the environmental monitoring of the Mar Menor coastal lagoon (Spain) and the monitoring of the land use of its watershed. It integrates heterogeneous data sources ranging from ecological data obtained from a multiparametric oceanographic sonde to agro-meteorological data from IMIDA’s network of stations or hydrological data from the SAIH network as multispectral satellite images from Sentinel and Landsat space missions. The system is based on free and open source software and has been designed to guarantee maximum levels of flexibility and scalability and minimum coupling so that the incorporation of new components does not affect the existing ones. The platform is designed to handle a data volume of more than 12 million records, experiencing exponential growth in the last six months. The tool allows the transformation of a large volume of data into information, offering them through microservices with optimal response times. As practical applications, the platform created allows us to know the ecological state of the Mar Menor with a very high level of detail, both at biophysical and nutrient levels, being able to detect periods of oxygen deficit and delimit the affected area. In addition, it facilitates the detailed monitoring of the cultivated areas of the watershed, detecting the agricultural use and crop cycles at the plot level. It also makes it possible to calculate the amount of water precipitated on the watershed and to monitor the runoff produced and the amount of water entering the Mar Menor in extreme events. The information is offered in different ways depending on the user profile, offering a very high level of detail for research or data analysis profiles, concrete and direct information to support decision-making for users with managerial profiles and validated and concise information for citizens. It is an integrated and distributed system that will provide data and services for the Mar Menor Observatory.

## 1. Introduction

Coastal lagoons are critical ecological and socioeconomic ecosystems [1] (Appendix A) characterized by their shallow depth and high human irradiation [2]. The coastal lagoon of the Mar Menor is one of the largest coastal lagoons of the Mediterranean and is located in the Region of Murcia (Spain). It is a hypersaline and fragile ecosystem connected to the Mediterranean Sea through five channels with a maximum depth of fewer than 10 m. The main channel is the Rambla del Albujón, which significantly impacts the generation of runoff and sediments in the Mar Menor. This fact is demonstrated in the last episodes of torrential rains such as those that occurred: on 16–17 October 2003, in which intensities of more than 62 mm/h were recorded, the 50 mm/h in the episode of 15–19 December 2016, the 91 mm/h measured in the episode of 12–13 September 2019 or the 84 mm/h of 3 December 2019.

According to the Intergovernmental Panel on Climate Change (IPCC), one of the consequences of climate change is the increase in the frequency and intensity of torrential rains in many regions of the planet [3]. In the study area, events of this type are generally caused by isolated depressions at high atmospheric levels (DANA). This fact, together with the loss of the natural hydrographic network that traditionally existed in the watershed basin of the Mar Menor [4], causes an increase in nutrient and sediment inputs due to the dragging of water from agricultural and urban areas of the Campo de Cartagena to the Mar Menor. These inputs cause severe imbalances in the ecological state of its waters, causing massive phytoplankton blooms and anoxia events such as those that occurred in October 2019 after the torrential rains in September 2019 [5]. In addition, this type of coastal lagoon is threatened by extreme weather events such as heat waves [6]; in particular, in the Mar Menor, during 2022, there has been an increase in the average temperature of 4 °C, having exceeded the average temperature by more than 30 °C from July 18 to 14 August 2022.

On the other hand, by Decree 42/2021, of 31 March, of the Autonomous Community of the Region of Murcia, the “Integrated Management Strategy for Coastal Areas of the Socio-Ecological System of the Mar Menor and its Surroundings” was approved. This strategy establishes as an objective the creation of the Observatory of the Mar Menor (OMM), whose functions include coordinating the control and monitoring of the ecological state of the Mar Menor.

Coastal observatories that integrate different technologies are spreading in several countries in our environment to monitor eutrophication processes; some examples are the coastal observatory in the bay of Kalloni Bay in Greece [7] or the one developed in the Gulf of Trieste by the MED TOSCA project in Italy [8].

Space missions, such as Sentinel and Landsat, are revolutionizing how we understand and plan for more sustainable use of valuable land and soil resources. From urban planning, transportation routes and green spaces to precision agriculture, forestry and environmental management. These missions provide detailed and timely earth observation information to support decision-making. In addition, they provide high temporal and spatial resolution imagery with comprehensive coverage, and support the monitoring of agriculture, land use and land cover change, as well as coastal and inland waters; they even provide biophysical data, such as those related to chlorophyll and water content in leaves, or those linked to water quality indicators such as chlorophyll a concentration, cyanobacteria density or turbidity, among others [9].

The digitization process affects all productive sectors, and this technological revolution means starting to work differently, incorporating all the possibilities offered by Information and Communication Technologies (ICT) for managing large volumes of data being generated. The vast amounts of actual data being collected and managed are often being wasted because existing mechanisms cannot handle the complexities of this new scenario. All innovations are powerful on their own, but they are even more so when intertwined with applications that leverage data to enable managers to make more responsive and intelligent decisions. Artificial Intelligence (AI) and its particular applications, such as Machine Learning (ML), are proving to be very adept at detecting the many inefficiencies in modern society that contribute to ecosystem instability [10]. Another critical element in this process is the sheer volume of devices at our disposal, i.e., those objects with connectivity equipped with sensors, applications or other technologies that allow us to transmit and receive data to and from other things.

Managing large amounts of data has been a complex problem for years. Obstacles often arise when storing the information in a consistent and scalable system [11]. In addition, data tend to be organized in individual solutions that are not fully accessible to other systems. These separate sets of information create islands of unrelated information that make integration with other applications demanding.

Due to the number and variability of the data available on the Mar Menor and its watershed basin, as well as its exponential growth over time, we can be sure that we are dealing with a Big Data system. For this reason, difficulties arise in developing mechanisms to generate the best possible decision. This voluminous and variable data set cannot be managed with conventional data processing software. With the emergence of new technologies and software methodologies, this massive volume of data can be used to solve previously intractable problems. The important thing is not how much data we have but what we do with it. The data must be analyzed to obtain information that allows us to make the best decisions and to be able to prevent anomalous situations.

The motivation for this work is the need of the Regional Government of Murcia, Ministry of Water, Agriculture, Livestock, Fisheries, Environment and Emergencies to have a system that is capable of integrating all the available information on the Mar Menor coastal lagoon and its watershed to generate helpful information to make the best decisions for its conservation. The dissemination of this work will contribute to increasing the number of users of the system and possibly integrating new data sets from the monitoring area. For this reason, this work aims to propose a solution that collects massive data that provide information on the Mar Menor’s ecological state and its possible causes. The data sources are diverse: real-time data from permanently installed sensors, data collected manually through measurement campaigns, or data provided by satellite images. For this purpose, all the information collected is unified in a single and common data model that allows transforming the data into information. This transformation process must use classical techniques such as representation through groupings or graphics. However, it must also incorporate the most avant-garde techniques such as linkage to geospatial features and application of Machine Learning techniques, which represent this information differently depending on the end user. The proposed solution must be flexible and scalable enough to integrate new datasets easily. The proposed solution is based on free open-source software to reduce implementation and maintenance costs. The proposed solution provides data and services for the Mar Menor Observatory. This system is a living system whose purpose is to continue incorporating new data sets based on the technicians’ criteria to generate an early warning system based on filters defined to detect anoxia spots or fluctuations in the rest of the parameters.

The criteria chosen for bibliographic references are set out in Appendix A.

## 2. Study Area

**Figure 1:**
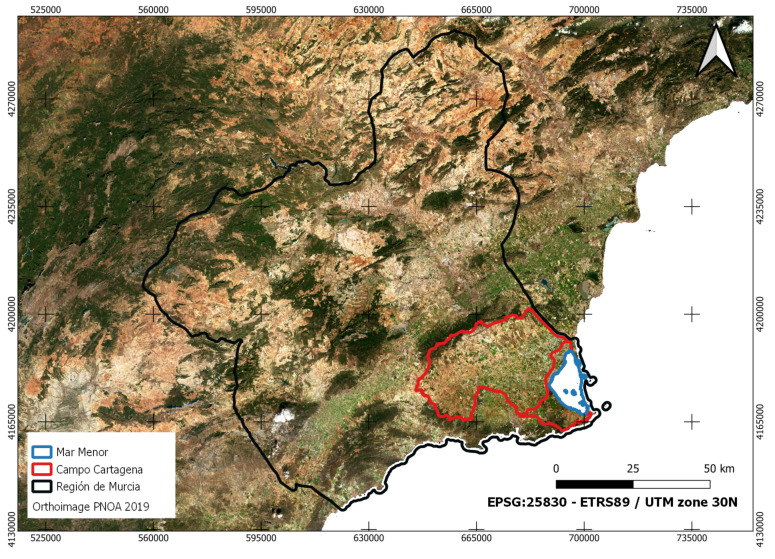
Study area in the Autonomous Community of the Murcia Region.

The Mar Menor is a saline coastal lagoon that occupies 135 km^2^ of surface, its volume is 653 Hm^3^, and its maximum depth is 7 m. It is located in the Campo de Cartagena, occupying an area of 1609 km^2^, of which 121,555 ha discharge their runoff into the Mar Menor, as shown in Figure 1.

The area has an arid or semi-arid Mediterranean climate, characterized by hot and dry weather. Based on data from the network of automatic stations of the Agricultural Information System of the Murcia Region (SIAM) [12] of the Institute of Agricultural and Environment Research and Development of Murcia (IMIDA) [13], it is determined that the average annual temperature of the area varies between 18 and 23 °C: the average annual rainfall during the last years is around 250 mm per year and usually occurs during autumn storms.

The Campo de Cartagena has a small plot size that gives the landscape a peculiar character, diversified and changing according to market demand, with crops of significant seasonal variation, which reach three annual rotations. The role of groundwater in this area is critical, especially in periods of drought or low water supply from the Tajo-Segura Aqueduct.

The base map in Figure 1 includes the most current image of the the National Plan for Aerial Orthophotography, also known with Spanish acronym PNOA [14], which for the study area of this work corresponds to the year 2019.

## 3. Data and Equipment

We have created a distributed framework for sharing large volumes of heterogeneous information for use in monitoring the Mar Menor. To this end, data analysis methods have been adapted and created for systems with Big Data requirements, including data representation, feature selection and modeling, to improve the accuracy and reduce the computational time of prediction algorithms to see how different elements of the environment affect the lagoon.

The data used by the platform proposed in this work come from different origins. Based on their origin, the data were classified as own, third-party, and remote sensing. The data from own sensors come from IMIDA and the Autonomous Community of the Murcia Region (CARM) [15]. Those third-party sensors originate from the Automatic Hydrological Information System (SAIH) [16] of the Segura Hydrographic Confederation (CHS). Finally, remote sensing-related data come from the European Space Agency (ESA) [17] and the National Aeronautics and Space Administration (NASA) [18]/U.S. Geological Survey (USGS) [19], through the Sentinel-2 and Landsat 8 and 9 missions.

Focusing on the typology of these data, we find biophysical, nutrient, satellite, piezometric, meteorological and hydrological data.

Table 1 shows a summary of the data used in the proposed platform, specifying indicators such as origin, type, periodicity or the identification code for subsequent reference, among others.

**Figure 2:**
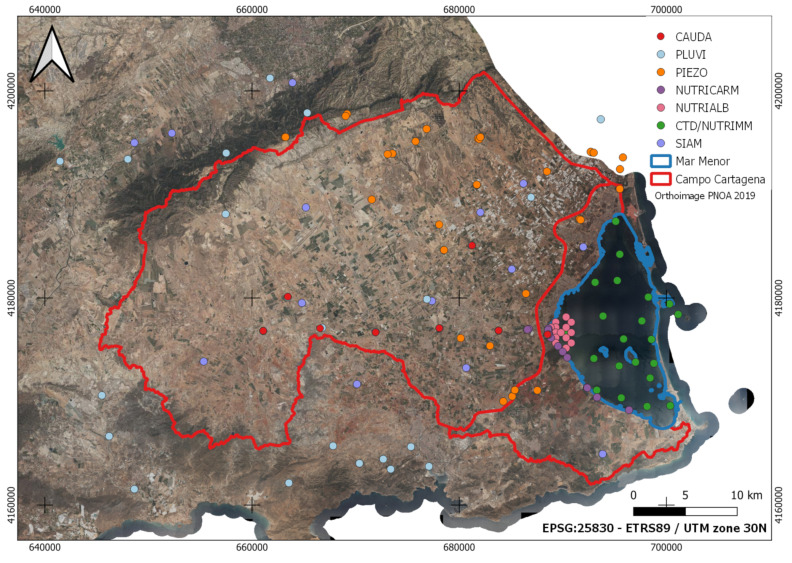
Distribution of input data in the study area.

Spatial information is essential in simplifying and illustrating environmental information and data in any ecosystem. In addition, it allows the investigation of variations generated in the field and cross-referencing with multiple data layers to generate new knowledge at different plot, farm, regional or even state levels. Establishing a solid and valid database is very important for managers and allows a spatial view, which enables better decision-making at any time. Figure 2 shows the spatial distribution of the different input data in the study area.

Like Figure 1, Figure 2 uses a base map of the PNOA’s most current image of the year 2019 for the study area of this work.

Table 2 shows the volume of data, expressed in number of records, handled according to their typology.

**Figure 3:**
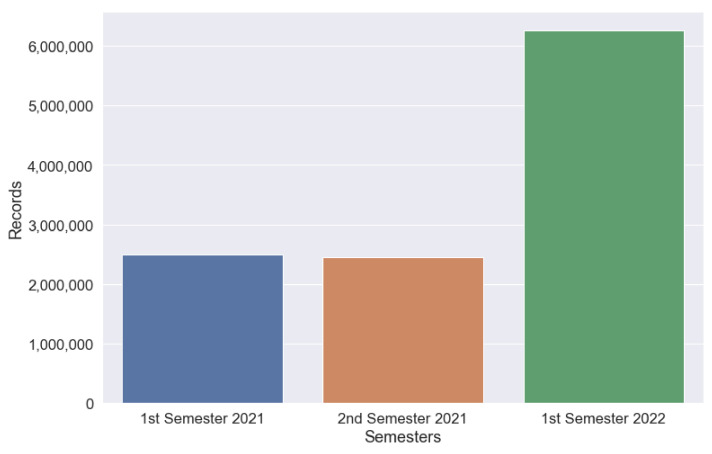
Evolution of the number of records processed by semesters, from 2021 to the first half of 2022.

Figure 3 shows the evolution of the volume of data, in terms of records, from January 2021 to June 2022. The exponential evolution seen in the above image is due to the continuous incorporation of new data sources.

### 3.1. Biophysical Data

In the case of monitoring the Mar Menor’s biophysical state, a dataset labeled CTD, weekly measurements are carried out on 21 predefined points on the water body of the Mar Menor. The time series goes from January 2021 to actual month 2022 (July).

**Figure 4:**
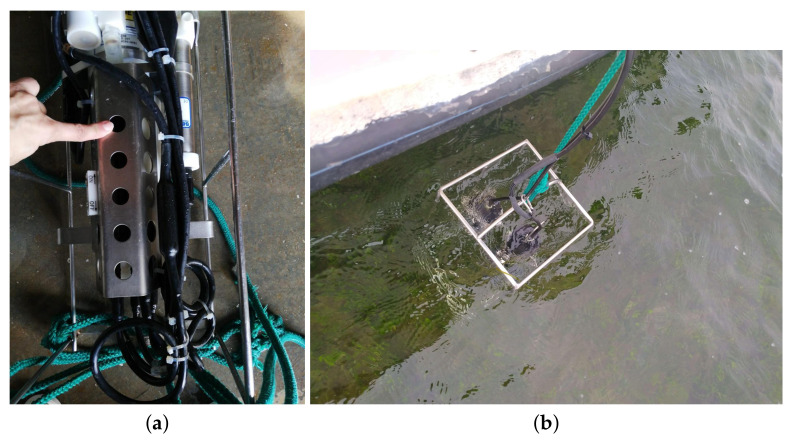
Oceanographic multiparameter sonde. (**a**) external view; (**b**) view taking data in the water.

For this purpose, IMIDA uses a SeaBird model SBE 19 plus multiparametric oceanographic sonde [20] equipped with temperature, conductivity and dissolved oxygen sensors, a fluorometer to obtain chlorophyll-a and turbidity and a pressure sensor from which the depth is obtained. Additionally, the field team usually generates a field sheet with information regarding the date and time of data collection, measurement point identifier, light penetration in the water according to the Secchi’s Disk [21], depth indicated by the sonde, and wind speed and direction. In addition, they take a photograph of the water at the measurement point. Figure 4 illustrates different views of the multiparametric oceanographic sonde; these images have been taken by IMIDA technicians.

As for the data format, the oceanographic sonde provides the data in a plain text file in cnv format. This file contains generic information about the measurement, such as date and time, battery and sensor status or calibration information, and the readings taken during the dive, which are usually around 500 records for each of these dives. In order to obtain these files, the sonde is connected to a computer with the sonde manufacturer’s application installed and downloaded. The field sheet data are recorded on a tablet and stored in ESRI geodatabase file format through the ArcGIS Survey123 tool [22]; this information is stored in the ESRI Cloud.

Additionally and extraordinarily, oxygen measurements are performed as it is one of the parameters that best reflects the biophysical state of the lagoon. The measurements are made a few meters from the shore and bordering the entire lagoon. The equipment used is a Hach HQ Series DO digital oximeter measuring 0.1–20.0 mg/L (ppm), saturation from 1 to 200%. This extraordinary measurement is also intended to complete the historical series. These campaigns are activated in the summer since there is a direct relationship between oxygen and water temperature [23] and are carried out daily. The mechanism used for the data collection of these campaigns is the same as that of the field sheet of the weekly measurements, i.e., using a tablet that stores the data in the ESRI Cloud in an ESRI geodatabase File through the ArcGIS Survey123 tool.

### 3.2. Nutrients Data

Regarding nutrient data, three different data sets are obtained. All three provide information on four parameters: ammonium, nitrites, nitrates and phosphates. The periodicity of data collection is weekly.

Water samples are taken to analyze nutrient levels. Specifically, the concentration of ammonium, nitrites, nitrates and phosphates will be determined. Since some of these compounds are very labile, most analyses will be carried out on the collection day to ensure no losses.

Ammonium concentration is determined by the indophenol blue method, proposed by Riley [24] and modified by Strickland and Parsons [25]. The process is carried out in an alkaline medium, and in the presence of nitroprusside, which acts as a catalyst, the ammonium ions treated by a solution of sodium hypochlorite and phenol give indophenol blue amenable to colorimetric determination.

The concentration of nitrite and nitrate is analyzed by the sequential determination method described by Garcia-Robledo [26]. This method is based on determining nitrites through the Griess reaction and the subsequent determination of nitrates after their reduction to nitrites with vanadium chloride.

Phosphate concentration is determined by the ascorbic acid method developed by Murphy and Riley [27]. The process takes place in an acid medium, and in the presence of ammonium molybdate, the orthophosphates form a phosphomolybdic complex which, reduced by ascorbic acid, develops a blue coloration amenable to colorimetric determination.

As for the location of the points where the measurements are taken, the NUTRIMM dataset shares the exact locations as the CTD dataset, i.e., 21 points spread over the entire surface of the Mar Menor water body. The NUTRIALB dataset has 15 points strategically located at the mouth of the Rambla del Albujón. NUTRICARM has 14 measurement points distributed between the Rambla del Albujón and its mouth and the riverside municipalities located south of the lagoon.

Regarding the format of these data, in the three data sets, the information is stored in the cloud, in plain text format with a specific separation character.

### 3.3. Meteorological Data

In order to determine the climatic conditions and their influence on the study area, agrometeorological information is collected from the SIAM, a service that is part of IMIDA. The SIAM was created to disseminate agrometeorological information from the network of automatic stations located in irrigable areas, as well as reports prepared from these data such as irrigation or cold and fertilization needs of the most widespread crops in the Murcia Region.

The stations are installed in irrigable areas, mostly on private farms, and must meet the requirements for their location defined by the technicians of the Regional Agricultural Offices. The resolution of the measurements taken by the stations is every five seconds, and the data are then stored on a ten-minute, hourly, daily, weekly, monthly and annual basis.

The service has 14 Campbell CR1000 automatic stations in the study area that collect data using GPRS communication protocols. The stations have measurements of maximum and minimum temperature (thermometer), maximum and minimum relative humidity (psychrometer), rainfall (pluviometer), wind (anemometer), radiation (radiometer) and evaporation; Table 3 describes the sensors of the stations. The stations are installed following the guidelines of the UNE 176101:2010 Standard “Networks of automatic agrometeorological stations. Characteristics, instrumentation and specific aspects”, which modifies Standard UNE 500520:2002 [28]. The agrometeorological records are processed using proprietary software and stored in a database. The selected stations have a historical series of about 25 years.

After receiving the data, they are validated by a procedure based on the UNE 500540:2004 “Automatic weather station networks: Guidelines for the validation of meteorological records from automatic station networks. Real-time validation”, which defines six levels of validation for data reliability. An explanation of this process can be found in Section 3.3.1. “Data Validation Module” of the work of Lopez-Morales et al. [29].

The data from these stations are dumped in the open data portal of the Murcia Region in real-time [30] so that any citizen or company can have access to a detailed climatological map of the Murcia Region. In addition to the historical series for each agroclimatological station, the open data portal offers the geolocation of each data in time ranges and with calculated data.

### 3.4. Hydrological Data

The SAIH comprises a set of technological infrastructures whose purpose is to capture, transport, process, distribute, archive and store real-time hydrological, hydraulic and complementary information for the entire Segura river basin. For the study area of this work, the SAIH provides 55 sensors that offer data for different purposes related to hydrology.

#### 3.4.1. Piezometric Data

Campo de Cartagena is influenced by the Quaternary multilayer aquifer consisting of 20–150 m of gravels, sands, silts and clays deposited on tertiary marls that act as an impermeable base, being one of the principal aquifers of the Mediterranean basin, in terms of extension, use and productivity of groundwater [31].

Since the 1970s, different state agencies have been monitoring the evolution of groundwater through a quantitative status monitoring network. This network is mainly composed of piezometers, which are small diameter boreholes precisely drilled to measure the water level in the aquifer, also known as piezometric level—the variable controlled in the piezometers in the water depth. The altimetric position of the water (piezometric level or surface) is a direct indicator of the water mass stored in the aquifer and of the characteristics of the flow within it. The piezometric level is, therefore, a key parameter for evaluating and managing the available water resource by providing descriptive historical series of its evolution, with which the conceptual models of groundwater flow are validated.

Through the SAIH, the CHS has a piezometric network that provides variables such as temperature, salinity, conductivity or dissolved solids. It also provides information on depth, which, together with the elevation (installed in the piezometer), allows us to know the height of the aquifer. Within the study area of this article, there are 20 stations of the piezometric network.

As for the information format, the CHS does not offer any Application Programming Interface (API) [32] that provides a mechanism for obtaining data from the SAIH. The only existing way to obtain piezometric data are by manual navigation.

#### 3.4.2. Flow Data

The SAIH also provides the flow data used in the platform proposed in this work. In this case, there is only one parameter, the flow rate. With this data, we can know the contribution of water to the Mar Menor from the Rambla del Albujón.

Concerning the data format, we find the same casuistry in Section 3.4.1.

#### 3.4.3. Pluviometers Data

The SAIH pluviometers network data have been labeled as hydrological type in this work since they only provide information related to precipitation. In order to be included in the meteorology data set type, additional variables such as temperature, wind speed or humidity, among others, would have to be provided.

Regarding this data format, we find the same casuistry as in the two previous subsections.

### 3.5. Satellite Data

Through the Copernicus Program [33], ESA provides infrastructure for earth observation and has among its objectives to provide information to facilitate environmental management. The Copernicus Program consists of five space missions called Sentinel. Sentinel-2 [33] is aimed at monitoring the Earth’s surface utilizing high-resolution optical images. Sentinel-2 offers in each image 13 spectral bands that provide a new perspective of the Earth’s surface and vegetation, with a resolution of between 10 and 60 m. The revisit period of Sentinel-2 is five days.

On the other hand, in collaboration with the United States Geological Survey (USGS), NASA offers the Landsat program, which includes the Landsat 8 and Landsat 9 satellites [34,35]. Among the main applications of this program is the observation of land cover change and land use, agriculture, hydrology, coastal resources and environmental monitoring, among others. The Operational Land Imager (OLI) sensor carried by these satellites can produce medium-resolution optical images. Through their OLI sensor, both Lansat 8 and Landsat 9 offer 11 bands with resolutions between 30 and 100 m in each of their images. The revisit period of these satellites is eight days.

The information described above is used to introduce another type of data used in the solution proposed in this work. The satellite data are used both to observe in a “human” way the state of the land cover of the Campo de Cartagena and the cover of the Mar Menor, through the visible bands and, through the non-visible spectrum bands, to emphasize certain elements such as vegetation, chlorophyll or turbidity, among others.

**Figure 5:**
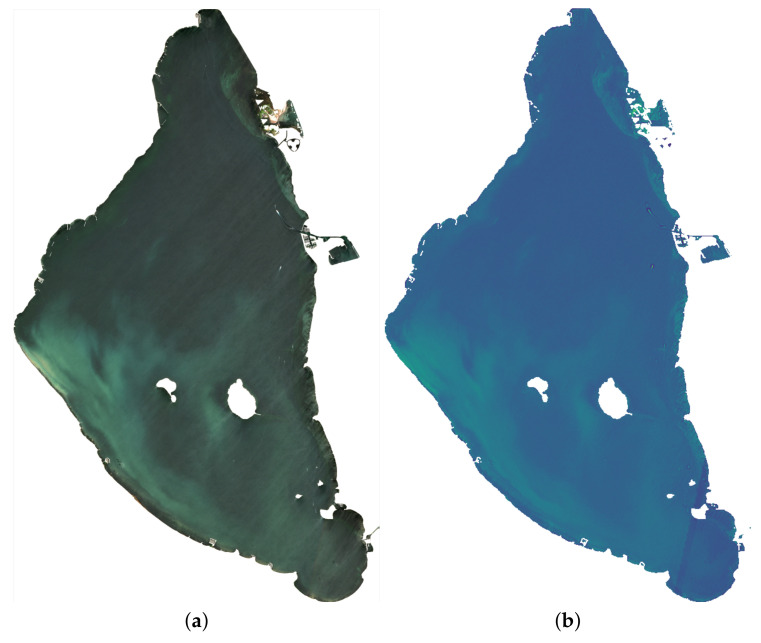
Sentinel-2 image of 9 June 2022, of the Mar Menor water body. (**a**) true color image; (**b**) image with turbidity index.

In Figure 5, the Sentinel-2 image of 9 June 2022 is illustrated in two different ways, as offered in the proposed solution. Figure 5a shows the image in true color, and in Figure 5b, the turbidity is enhanced through the index [36] that responds to the formula:

(1) $$\mathrm{Turbidity}=8.93*\frac{B03}{B01}-6.39$$

Concerning Sentinel-2 images, the S2MSI2A product with processing level 2A, BOA reflectivity and multispectral reflectance is used. On the Landsat images, Collection 2 Level 2 is used, whose main characteristics are the atmospheric correction and scientific products of reflectance and global surface temperature.

Regarding the format of these data sources, both have similar characteristics. They are offered as compressed files in ZIP format, and their contents include files in XML or JSON format containing the image metadata and other files in the form of geolocated images in JPEG2000 or Geotiff format.

This type of data is used to monitor around 100,000 plots in the Campo de Cartagena area through the vegetation index, to estimate the irrigation needs of the Mar Menor watershed and to have more indicators on the state of the lagoon.

## 4. Infrastructure

The starting point of this work consists of integrating heterogeneous and unconnected data sources that must converge and enable the data transformation process to provide different information based on each user’s profile. Based on these characteristics, it was decided to look for a flexible and decoupled infrastructure with different components or elements. Each component is connected through a shared interface but can work independently. Furthermore, the performance and operation of each component generally do not affect the performance of the other components. Finally, the infrastructure must be flexible enough to allow the addition of new components without affecting existing ones.

As for the software chosen for the deployment, the premise is to comply with low implementation costs; therefore, an open source option should be chosen. Furthermore, a Platform As a Service (PaaS) [37] architecture type is chosen, as it simplifies the productive operation of the applications and allows new components to be added quickly and easily. After analyzing virtualization technologies such as LXC, Docker or Kubernetes [38], it was decided to opt for Docker [39].

Docker is an Open Source project from 2013 that revolutionized how applications are developed and deployed, allowing applications to be separated from the infrastructure. It allows environments to be virtualized as it simulates a set of lightweight virtual machines without the need to reserve physical resources; an intermediate zone is created between the physical operating system and the virtualized system that provides an environment independent of the physical machine; these virtualized resources are called containers. Containers are used to deploy services such as database and web servers or in-house developed applications. Docker provides the flexibility to create containers, deploy, copy and move them from one environment to another, which facilitates scalability. Finally, Docker Swarm offers clustering capabilities [40], allowing a generation of a group of Docker virtual machines deployed in different physical machines and configured to be united and work in unison; the main advantages are the high level of availability, the high level of scalability or the load balancing options.

**Figure 6:**
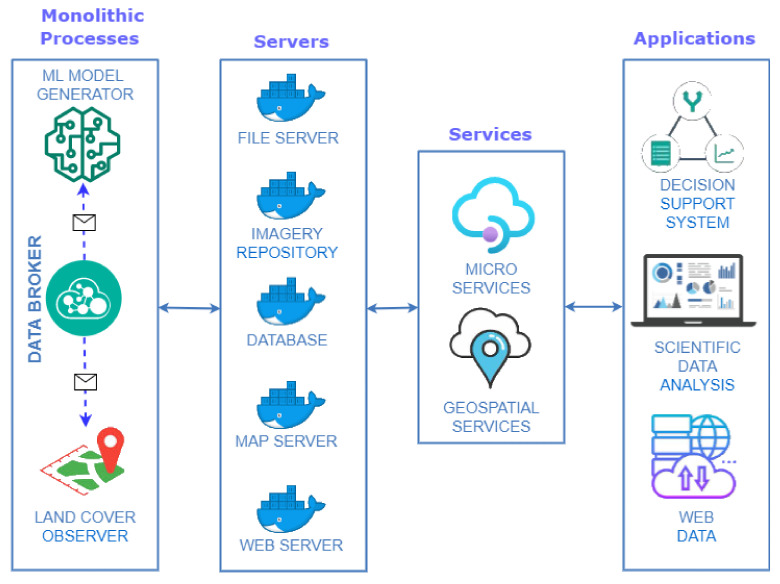
Diagram of the proposed infrastructure.

Figure 6 illustrates the infrastructure proposed in this work in a summarized way. Each element in this figure translates into a Docker container with a specific configuration.

Focusing on the purpose of the components shown in Figure 6, the following grouping is made:

- Monolithic processes: This combines the components responsible for generating input information to the system differently. Communication between its components is done through a publish/subscribe system. A publish/subscribe system connects these components. A publish/subscribe system is a universal many-to-many communication paradigm that provides a scalable and adequate interaction scheme, guaranteeing the dissemination of new events to subscribers who have expressed interest in the same event [41].
- Servers: This encompasses the components responsible for persisting incoming information in different media to remain available and accessible over time.
- Services: Includes the components responsible for the creation of microservices [42]. Microservices are an architectural and organizational approach to software development, where software is composed of small, independent services that communicate through APIs [43]. Each service performs a single function, and because they run independently, each service can be updated, deployed and scaled.
- Applications: Components that translate into applications with different levels of access display different information depending on the type of user.

The infrastructure deployment is done on a proprietary server with an Intel Xeon Gold processor, 128 GB of RAM and an NVIDIA Tesla V100 graphics card. The operating system chosen is CentOS Linux 8.

## 5. Proposed Solution

This work aims to integrate different heterogeneous datasets related to the Mar Menor and its catchment area and persist them in an information model, transforming them into information that can be represented differently according to the user’s needs.

The instantiation of the proposed infrastructure is based on free and open source software. Python and JavaScript have been used as the primary programming languages. A PostgreSQL database with the PostGIS spatial extension is used for data persistence. The web applications have been developed with the Python Streamlit library [44] and the JavaScript runtime environment NodeJS [45].

The characteristics of each element in Figure 6 that make up the platform for improving decision-making for the Mar Menor, and the elements that affect it are detailed below.

### 5.1. Servers

Although in the infrastructure proposed in Section 4, this category is in the second position, it is necessary to talk about it first since these infrastructure elements support the rest of the elements of the proposed solution.

**Figure 7:**
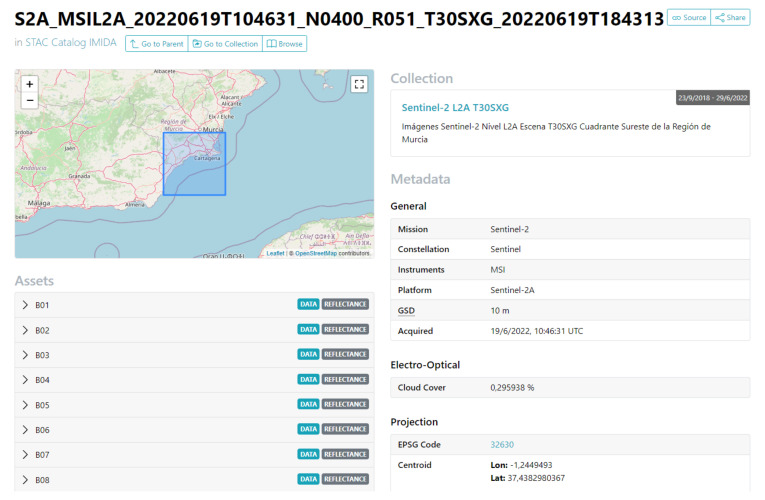
Example of the metadata screen of the T30SXG scene of 19 June 2022 in the STAC Browser.

In this work, four servers with different purposes are deployed, and these components are instantiated as a Docker container. As a reference, Figure 6 specifies which type of technology is instantiated concerning the servers of the proposed infrastructure:

- File Server: in charge of storing files and making them accessible for use by the rest of the components. The information stored in this element includes satellite images and derived products that may be generated, photographs that may be taken in in-situ measurement campaigns or serialization of models generated through Machine Learning, among others. The product selected to carry out this task is LocalStack [46], a cloud services emulator running in a single container.
- Imagery Repository: responsible for arranging the metadata of satellite images and their derived products (indexes, mosaics or other elements). A structure of files and directories forms this infrastructure element according to the SpatioTemporal Asset Catalog specification (STAC) [47,48]. This specification is a language for describing geospatial information, making it easier to discover, work with and even index. Concerning STAC, a browser of this type of catalog is deployed to access the images, their metadata and their bands easily [49], as can be seen in Figure 7.
- Database: in charge of persisting the large amount of data to be handled, using a database management system, such as PostgreSQL. In addition, to provide it with geospatial characteristics, the PostGIS extension is added.
- Map Server: offers the geographic information associated with the data processed and generated by this work, services that implement the OGC (Open Geospatial Consortium) standard [50] must be generated. In the proposed solution, only Web Map Service (WMS) type services are published, which only offer the possibility of visualizing the spatial dataset. The GeoServer product is responsible for performing these tasks.
- Web Server: responsible for distributing and delivering Web content and responding to Web requests. Therefore, this type of server will be used both for publishing the applications proposed in this work and for deploying the microservices. The product selected to carry out this task is Nginx.

### 5.2. Monolithic Processes

A monolithic process is self-contained and independent of other processes, encompassing the user interface layer and the logic and data access layers. In addition, they can perform all the tasks necessary to complete a given function and are designed without modularity. Monolithic processes are used because they are tightly coupled to the rules of the proposed system domain by being autonomous and independent of other processes.

In this section, we will detail the components of the proposed solution that have a monolithic character and are executed independently from the rest of the elements.

#### 5.2.1. Data Broker

While each component is essential, the Data Broker is vital because it is responsible for supplying data to the system and orchestrating other components to carry out its task. Its general purpose is to collect data from different sources and, through ETL (extract, transform and load) techniques [51], persist the data in different media. All retrieved data have an associated geographic component that allows them to be located on a map.

To orchestrate the launching of the different sub-processes, the container itself performs a series of scheduled tasks executed with a certain periodicity.

**Figure 8:**
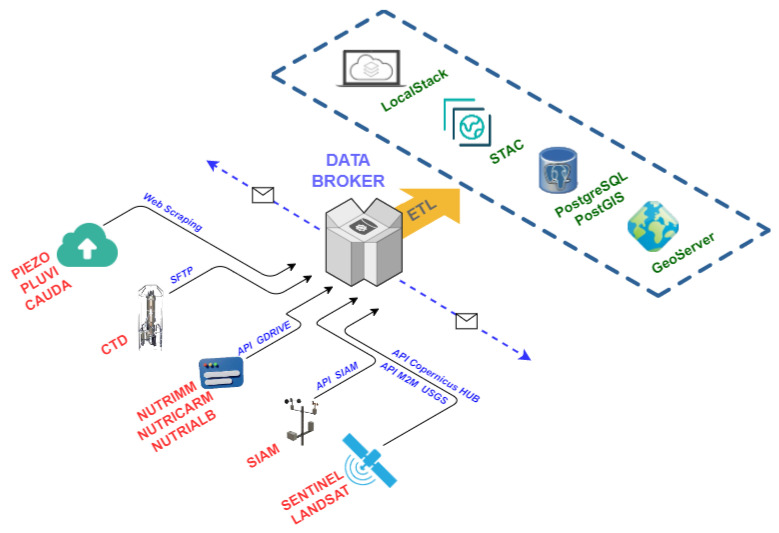
Workflow of the Data Broker component.

Figure 8 represents the workflow linked to the Data Broker component.

Different techniques are used to obtain the data depending on the input dataset. Thus, the Google Drive API is used for nutrient data sets (NUTRIMM, NUTRIALB or NUTRICARM). For CTD data, a Secure File Transfer Protocol (SFTP) communication is used, and for SIAM data, the Open Data API of this service is used. For SAIH data (PIEZO, PLUVI and CAUDA), Web Scraping techniques are used as there is no API for this purpose, and, finally, for satellite images, use is made of the corresponding APIs, which are OpenSearch API for Sentinel [52] and Machine-to-Machine (M2M) API [53] for Landsat images.

Data persist in four different ways, alphanumeric and geospatial information in the PostgreSQL+PostGIS database, physical files such as photographs from field campaigns or satellite imagery in the LocalStack-based file server, satellite image metadata in a STAC, and geospatial information, either raster or vector format, in GeoServer.

Once the Data Broker has performed all operations, it sends notifications to the ML Model Generator and Land Cover Observer components to perform the assigned processes. The ML Model Generator and Land Cover Observer components are subscribed to Data Broker through a WebSocket communication [54]. Through this communication channel, information is sent in serialized JSON format [55].

This element is implemented with Python and uses ETL libraries, such as pandas or numpy, satellite data manipulation libraries such as rasterio or GDAL, geopandas or fiona for working with vector geographic information or psycopg for persistence in the PostgreSQL database.

#### 5.2.2. Land Cover Observer

This component is in charge of monitoring the Campo de Cartagena area’s land cover to locate crop areas. For this purpose, geographic information systems and remote sensing are being used, specifically the multispectral and multitemporal analysis of crops, an analysis that includes, for each date, the analysis of active vegetation and the determination of crops under plastic or crop cycles number.

This component relies on satellite images, preferably Sentinel-2 [56], for their higher spatial resolution, and combination with vector data through spatial analysis operations. For this, it is necessary to have products derived from the images, such as spectral indices and, in our case, vegetation indices. These indices are combinations of the spectral bands of satellite images whose function is to enhance the vegetation cover and attenuate the details of other components such as soil. For this reason, these indices can be considered images calculated from algebraic operations between different spectral bands.

In this work, we have used the Normalized Difference Vegetation Index (NDVI) [57], which responds to the formula:

(2) $$\mathrm{NDVI}=\frac{\mathrm{Near}-\mathrm{Infrared}-Red}{\mathrm{Near}-\mathrm{Infrared}+Red}$$

The range of possible values is from −1 to 1. Negative values are associated with areas of water and snow, and positive values close to 0 represent rocky and bare areas that may acquire some vegetation up to values close to 0.3. From this value onwards, we find the presence of vegetation. The higher the value, the more lush the vegetation until values close to 1.

**Figure 9:**
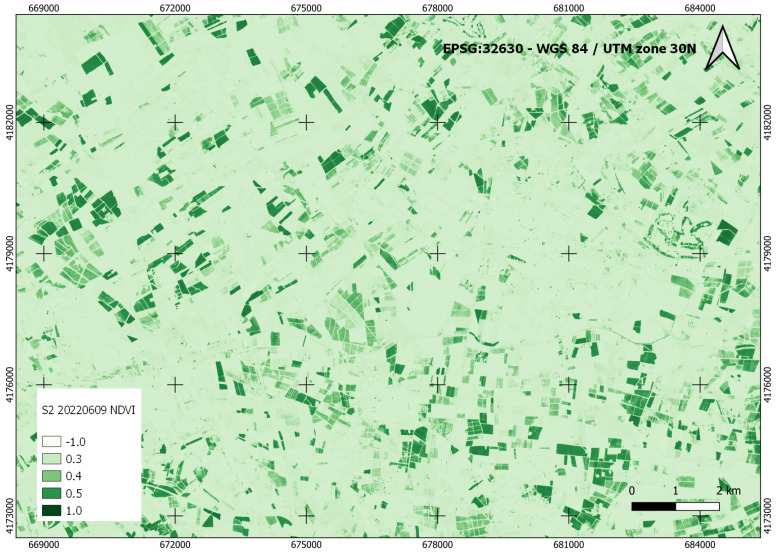
NDVI index of a zone of the study area from the Sentinel-2 image of 9 June 2022.

Figure 9 shows the NDVI index generated for a zone of the study area from the Sentinel-2 image of 9 June 2022. The color scale has been set to emphasize the vegetation zones.

Through remote sensing, this product is intended to monitor land use, obtain an estimate of the area cultivated in the watershed at any time in the time series and determine the number of crop cycles.

As indicated in Section 4, the Land Cover Observer component is connected to the Data Broker through a publish/subscribe system. In practice, there is a communication channel between these two elements; when Data Broker downloads a valid satellite image for the study area, it sends a notification to Land Cover Observer indicating that a new image is available. This notification is translated into a serialized JSON file in the format of Listing 1.

**Listing 1:** JSON example notification between Data Broker and Land Cover Observer for S2 image.

{ 
   " mission ": "S2", 
   "id": " 9837721f -2 d51 -4014 - b2fb -7 d3fa6f06207 ", 
   " date ": "2022 -06 -09" , 
   " name ": " S2A_MSIL2A_20220609T104631_N0400_R051_T30SXG ", 
   " cloudcover ": 1.029, 
   " footprint ": " MULTIPOLYGON ((( -0.644 36.934 , -0.613 
   37.923 , -1.861 37.942 , -1.876 36.952 , -0.644 36.934) )) 
   ", 
   " extrainfo ": " L2A_T30SXG_A036370_20220609T105816 " 
}
		  


The following is the meaning of each of the keys that appear in a satellite image download communication JSON:

- mission: space mission to which the image belongs. It can contain the values S2 (Sentinel-2), L8 (Landsat 8) and L9 (Landsat 9);
- id: internal identifier of the image. It is used to locate the metadata registered in the database;
- date: date of the image;
- name: image name with additional information such as orbit or scene can be found;
- cloudcover: percentage of cloud cover;
- footprint: image coverage area;
- extrainfo: additional information that may be useful for image processing.

**Figure 10:**
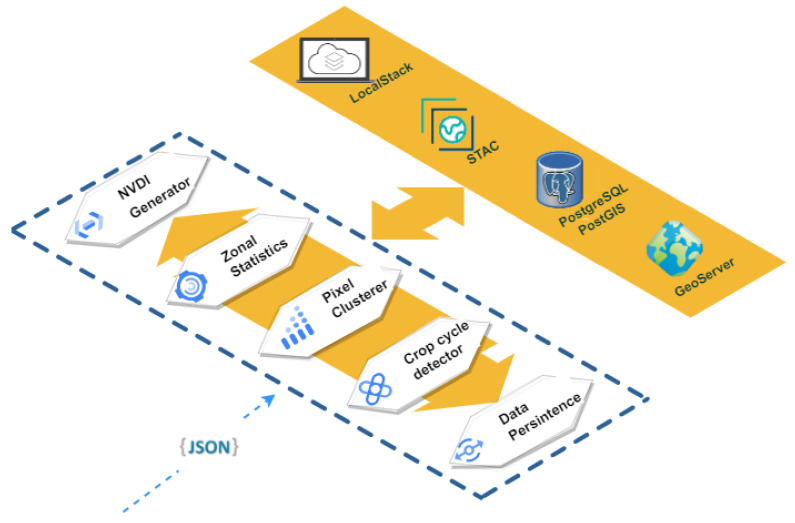
Workflow of Land Cover Observer component.

Figure 10 illustrates the workflow of the Land Cover Observer component.

The atomic tasks carried out in the Land Cover Observer component, once it has received the notification from the Data Broker, are detailed below:

1. NDVI Generator: the information is obtained from the corresponding satellite image in the STAC catalog. Once all the necessary information is obtained, the NDVI index is generated, applying the Formula (2).
2. Zonal Statistics: this task is in charge of crossing the Campo de Cartagena plots with the NDVI index generated in the previous step in order to obtain certain statistical data, such as mean, standard deviation or variance, for each of the plots.
3. Pixel Clusterer: atomic process in charge of grouping the pixels of each plot, according to the NDVI index value, in ranges of values. In this work, the pre-established ranges of values are those that can inform us which pixel is or is not cultivated. Specifically, the groups are values less than 0.1, between 0.1 and 0.2, between 0.2 and 0.3 and greater than 0.3. By knowing the pixel size of the satellite image, we also know the area of each of the ranges.
4. Crop Cycle Detector: based on the NDVI index, this subprocess is responsible for detecting the crop cycles in each plot in the Campo de Cartagena area. This process is based on a series of predefined NDVI thresholds, establishing the states of bare and cultivated soil [58].
5. Data Persistence: at this point, all the information generated by the previous points persists in different ways. Thus, the data generated by Zonal Statistics, Pixel Clusterer or Crop Cycle Detector are stored in the PostgreSQL database. Data generated by the NDVI Generator is stored in LocalStack and STAC. Furthermore, any generated data containing a geospatial component is published as a service in Geoserver.

This element is implemented with Python and is supported by libraries for treating and manipulating raster images such as rasterio, GDAL, xarray, stackstac or rasterstats. In addition, geopandas or fiona are used to work with vectorial geographic information, and localstack or psycopg are used for data persistence.

The Land Cover Observer component generates a time series for each agricultural campaign [59], which, in this case, covers the dates between September of the previous year and August of the current year. For example, the time series for the agricultural campaign 2022 would cover the dates between September 2021 and August 2022. In any case, the data from the different time series can be combined.

#### 5.2.3. Machine Learning Model Generator

The specific purpose of this component is to generate a Machine Learning model that allows predictions about the biophysical state of the Mar Menor. The element defined in the infrastructure as ML Model Generator, like the Data Broker element (Section 5.2.1), is a monolithic component that is executed independently each time the Data Broker collects all the input information for an entire week (Table 1).

Like the Land Cover Observer component (Section 5.2.2), the ML Model Generator element is subscribed to the Data Broker component through a direct and continuous communication channel. When Data Broker has retrieved all the weekly data from the CTD, SIAM, NUTRIMM, NUTRIALB, NUTRICARM, PIEZO, PLUVI and CAUDA datasets, it sends a notification to ML Model Generator in the form of JSON for it to execute. An example of this communication is shown in Listing 2.

**Listing 2:** JSON example notification between Data Broker and ML Model Generator.

{ 
   " startweek ": " 20220606" , 
   " endweek ": " 20220612" , 
   "ctd": [" 20220606 "], 
   " siam ": "all", 
   " nutrimm ": [" 20220606 "], 
   " nutrialb ": [" 20220607 ", " 20220610 "], 
   " nutricarm ": [" 20220609 "], 
   " piezo ": "all", 
   " pluvi ": "all", 
   " cauda ": "all" 
}
		  


The meaning of each of the keys that appear in the Machine Learning-based model generation communication JSON is detailed below:

- startweek: start date of the week;
- endweek: end date of the week;
- ctd: set of dates of the new data corresponding to the CTD dataset;
- siam: set of dates of the new data corresponding to the SIAM dataset. If the all keyword appears, it refers to the totality of the week’s dates;
- nutrimm: set of dates of the new data corresponding to the NUTRIMM dataset;
- nutrialb: set of dates of the new data corresponding to the NUTRIALB dataset;
- nutricarm: set of dates of the new data corresponding to the NUTRICARM dataset;
- piezo: set of dates of the new data corresponding to the PIEZO dataset. If the all keyword appears, it refers to all the week’s dates;
- pluvi: set of dates of the new data corresponding to the PLUVI data set. If the all keyword appears, it refers to all the week’s dates;
- cauda: set of dates of the new data corresponding to the CAUDA dataset. If the all keyword appears, it refers to all the week’s dates.

As input, the model receives the weekly average data given in the keys of the JSON file above. The model must solve a regression problem based on time series. The generated model finally gives a predicted value for the biophysical parameters of oxygen, chlorophyll, turbidity or salinity for each of the 21 points where samples are taken with the multiparameter oceanographic sonde.

**Figure 11:**
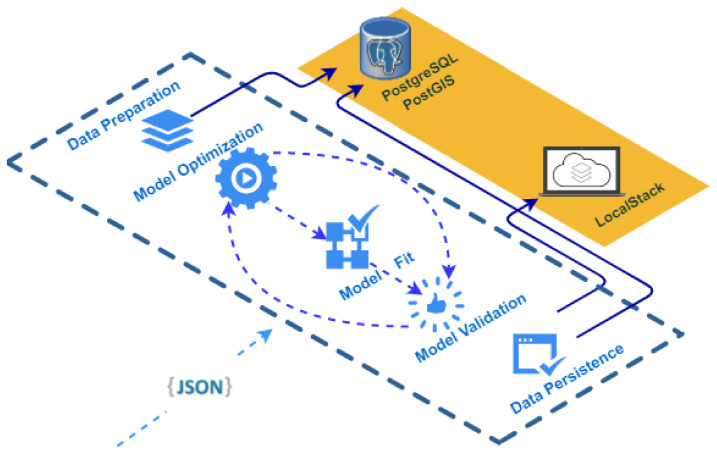
Workflow related to the ML Model Generator component.

The following is an explanation of the atomic tasks carried out in each of the elements of the ML Model Generator [60] element workflow, which can be seen in Figure 11:

1. Data Preparation: the input data required for the model are obtained and formatted so that the Machine Learning model can subsequently process them. To summarize, all the features or dependent variables must be numerical. Therefore, feature engineering techniques [61] are applied to, for example, transform data in date or categorical format to numerical format.
2. Model Optimization: in this step, a set of hyperparameters of the Machine Learning model are optimized through the Grid Search technique [62] and K-Fold cross-validation [63], with k = 10.
3. Model Fit: to fit a Machine Learning model, it is necessary to have a training matrix. In this case, the training matrix corresponds to the weekly average data coded as CTD, SIAM, NUTRIMM, NUTRIALB, NUTRICARM, PIEZO, PLUVI and CAUDA, which are collected with manual measurement campaigns in the field or collected by different sensors. For the separation of the training matrix between training and test, the Walk-Forward validation [64] method is used to respect the temporal order of the observations.
4. Model Validation: the fitted model is validated by obtaining different metrics such as Mean Squared Error (MSE), Root Mean Squared Error (RMSE) and Mean Absolute Error (MAE).
5. Data Persistence: in this last step, on the one hand, the results of the metrics analyzed in the validation process or the configuration of the hyperparameters of the model, among others, are stored in the database. On the other hand, the adjusted model is serialized in the LocalStack component to retrieve and load it when making predictions.

Steps 2, 3 and 4 are repeated as often as models are evaluated. The models that are evaluated in the ML Model Generator component are Random Forest [65], LightGBM [66] and XGBoost [67]. Thus, when making predictions, the best performing model, or a specific model, can be selected, and the metrics obtained during fitting can be considered.

This workflow is repeated weekly once all the input data for the corresponding week has been collected. Predictions over time become less and less accurate, and therefore it is a realistic approach to retrain the model with accurate data as it becomes available for new predictions. Since statistical training models do not require much computational time and effort, Walk-Forward Validation [64] is the right solution to obtain more accurate results.

The generated model is currently being validated with data taken in situ. For this purpose, a quality assessment is applied following the statistical assumptions of the binomial distribution underlying the international standard ISO document 2859/2 procedure B [68].

For the implementation of this module, an environment, based on the Python programming language and the Machine Learning library, scikit-learn, is used.

### 5.3. Services

For the development of the proposed solution, based on the development of a software application, an architecture whose main element is the microservices is used; each of the microservices is executed autonomously and communicates with each other through HTTP requests, in our case. Each independent service has a simple and concrete purpose and can be deployed without affecting the rest of the services.

**Figure 12:**
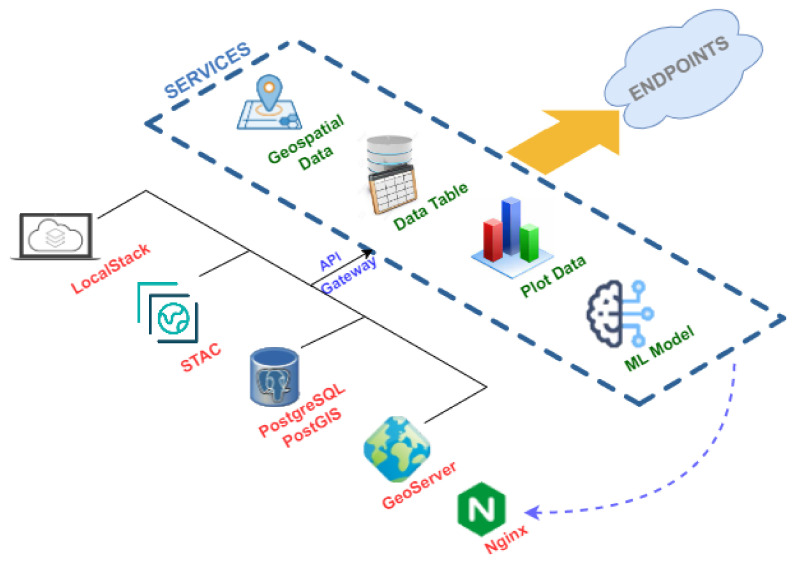
Service generation workflow.

In Figure 12, the workflow performed by the microservices component is shown.

Focusing on the type of output information it provides, we find four different types:

- Microservices that provide geospatial information, such as interpolations, vector layers, satellite imagery or products derived from these, whose output format can be an image in base64 [69] format or an OGC service in WMS format;
- Information in table format with alphanumeric information whose output format is JSON [70];
- Graphical information whose output format can be HTML or a base64 image;
- Information related to the Machine Learning model generated (Section 5.2.3), which can be the serialized model itself or the metadata stored in the database when generating the model, among others.

If the focus is on the organization of the different microservices, they are organized according to the data source typology. Thus, following the nomenclature in Table 1, we find the namespace CTD, SIAM or PIEZO, among others.

This is undoubtedly the most complex and essential component of the proposed infrastructure. Thus far, about 150 endpoints have been generated and can be accessed via HTTP since they are finally deployed on a Web server.

**Figure 13:**
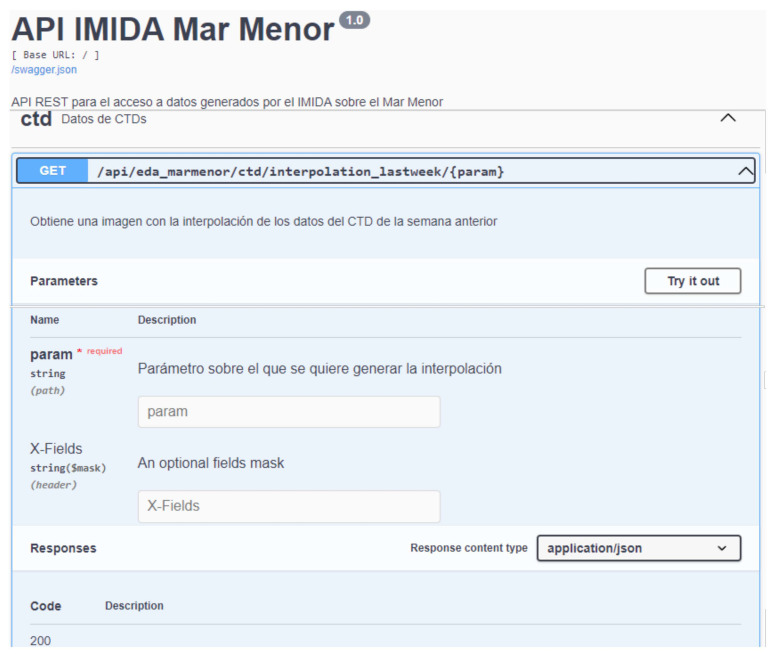
Example of interpolation endpoint Swagger for the week of 18–24 July 2022.

Finally, to facilitate their use by third parties, all the microservices have been documented following the Swagger guidelines, an open specification for API definition. Through this documentation, it is possible to consult the list of resources available in the API and the operations that can be called on these resources and provide an interface for testing the invocation of the defined endpoints. Figure 13 shows an example of how this documentation is provided.

This element is implemented in Python and uses the flask library and some of its extensions to generate microservices. For the graphical representation it uses matplotlib, plotly or seaborn. The representation of geospatial data are handled by gdal, rasterio or geopandas. For everything related to Machine Learning, the scikit-learn library is used.

### 5.4. Applications

Intelligent decisions can only be based on reliable data from integrating multiple sources into a single data set. To make optimal use of the available data, it is necessary to use appropriate transformation techniques to solve compatibility problems and thus improve data consistency [71]. The process begins by identifying the sources, extracting the data and transforming it into an understandable format, and verifying that it is accurate and of the quality required for further analysis. The last step is to clean up any erroneous data that may have been generated, and, finally, the data are unified, classified, aggregated and stored in the system.

The availability of massive volumes of data does not guarantee that they will be helpful for any specific purpose, and it is necessary to convert this data into useful information. For this reason, as shown in Figure 6, different applications are created according to the user’s profile and the type of information they can visualize. All these applications are based on the components described above and access the same data sources but can transform these data into information in one way or another depending on the end user’s needs.

Thus, we propose an application that generates a large volume of information of all types, with a low simplification of the data for users and with high parameterization options, aimed at users with a scientific or data analysis profile (Scientific Data Analysis). Another application is intended to support decision-making and is aimed at users with a managerial profile; in this case, information is offered with a medium simplification of the source data and with medium parameterization options, displaying this information in most cases in the form of a graph or image and offering facilities for its interpretation (Decision Support System). Finally, the third type of application is offered to the general public and offers highly simplified information, with no parameterization options and mainly based on consolidated and validated data (Web Data).

When a new set of input data or a new way of representing certain information is incorporated, it is first implemented in the Scientific Data Analysis tool, then in the Decision Support System and finally in the Web Data.

#### 5.4.1. Scientific Data Analysis

It is intended to help the scientist or data analyst to draw conclusions about the state of the saltwater lagoon and to be able to establish the causes of this state. To achieve this, it offers the actual data, in every conceivable form, with all kinds of complexity and extensive parameterization possibilities. When new input data are recorded in the persistence elements, they are immediately available and searchable. It is the element of the proposed solution on which new input data sets are initially incorporated, and new functionalities are implemented. Once tested in this application, it is decided whether and how to incorporate it into the other applications.

**Figure 14:**
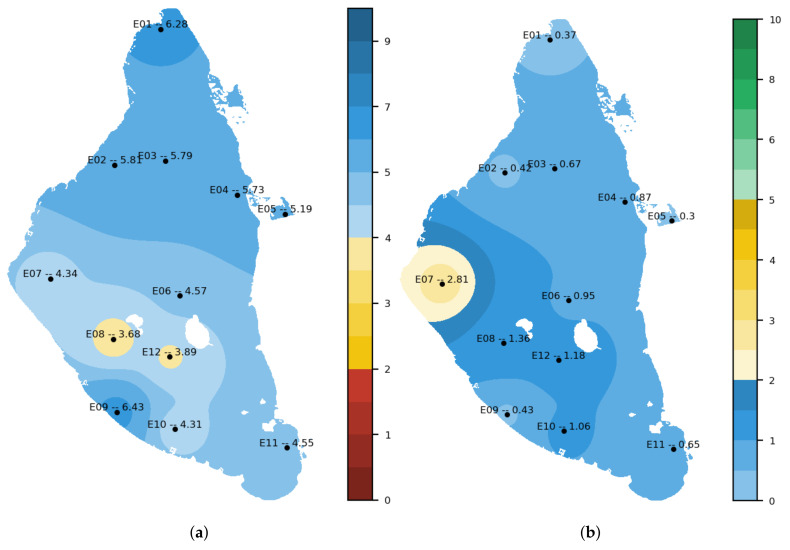
CTD data interpolation generated in Scientific Data Analysis application. (**a**) oxygen; (**b**) chlorophyll A.

The representation of the massive dataset is done through tables, graphs of various types of satellite images, and results of interpolation operations. Different interpolation methods, such as Inverse Distance Weighting (IDW) or Kriging [72], are offered. Figure 14 shows an example of oxygen and chlorophyll A interpolations from CTD measurements taken on 14 July 2021 offered by the application.

In addition, it allows the download of these representations in CSV format in the case of tables and image format in the case of graphs and images. In the case of satellite or interpolated images, the application generates georeferences to allow their later manipulation in a desktop GIS.

**Figure 15:**
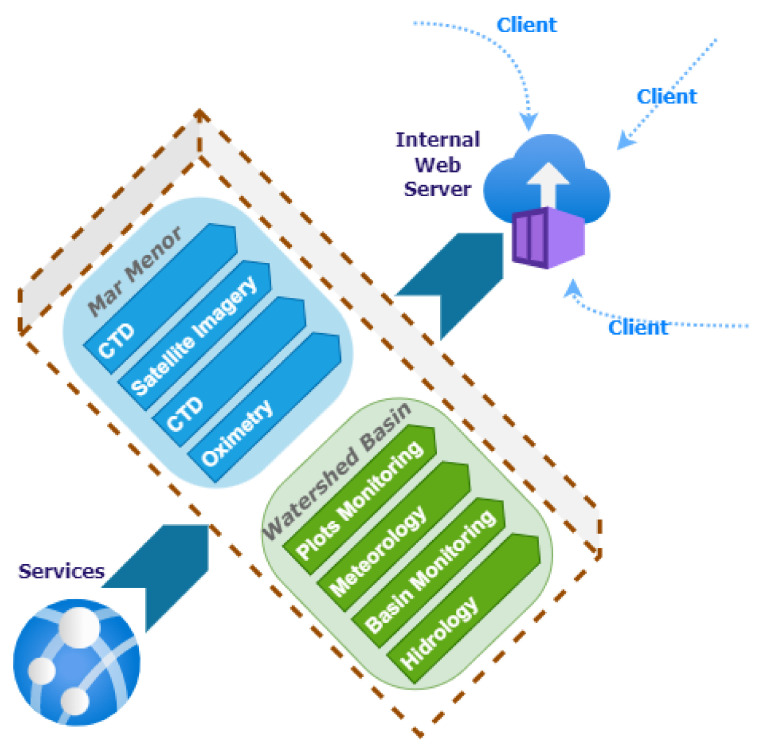
Diagram showing how the Scientific Data Analysis application works.

Figure 15 outlines how this application retrieves data and serves them, as well as the options available to the user.

According to the previous figure, the Scientific Data Analysis application offers the first option, the selection of the monitoring area. Depending on the monitoring area, the user finds different sub-options. These sub-options are organized according to the datasets treated as input (Table 1).

The application is deployed on an internal server, which is not accessible outside the organization and is implemented with Python and the streamlit library. Streamlit is a free and open source framework that offers the possibility of deploying data and Machine Learning applications quickly in a Web environment.

##### Mar Menor Monitoring Zone

When selecting the Mar Menor monitoring area, this application offers the CTD, Satellite Imagery, Nutrients and Oximetry options.

The CTD option offers all the data and information related to the CTD input dataset; this is the data collected with the measurements made with the multiparameter oceanographic sonde. It allows for obtaining these raw data for each measurement point and their graphical representation. It also allows data grouping by depth or location of the measurement point, among others. In addition, it offers the data represented globally in which the massive data can be grouped by the mean or by the geometric mean by water volume. The following formula is used to calculate the geometric mean:

(3) $$x=\frac{\sum_{i=1}^{n}x_{i}w_{i}}{\sum_{i=1}^{n}w_{i}}=\frac{x_{1}w_{1}+x_{2}w_{2}+...+x_{n}w_{n}}{w_{1}+w_{2}+...+w_{n}}$$

The weighting factor obtained by bathymetry in a previous survey [73] can be found in Table 4.

**Figure 16:**
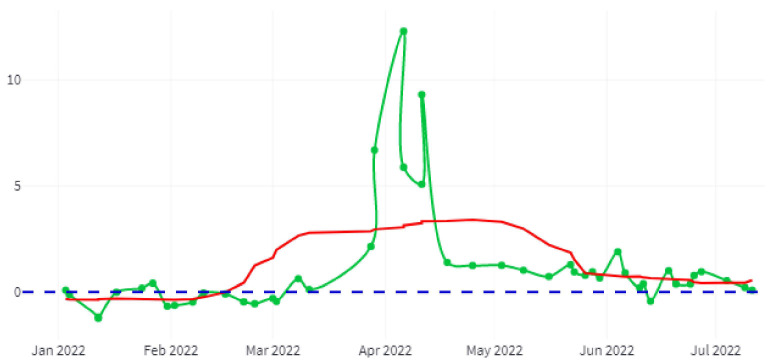
Annual comparison of turbidity between the year 2022 and the same month of the previous year.

Within the same CTD option, annual comparisons of the biophysical parameters included in the dataset can be obtained. In this comparison, it is possible to include adjustment curves of different types (moving average, polynomial, or others) fully parameterizable to detect trends. Figure 16 shows, in green, the difference between the turbidity status of the year 2022 for the same month of the previous year, measured in Formazin Turbidity Units (FTU) [74], together with the trend in red.

The Satellite Imagery option allows for accessing Sentinel-2 and Landsat satellite images and viewing them through different color compositions. If a natural color is desired, a series of filters can be applied to emphasize certain aspects of the image, such as histogram equalization or gamma adjustment [75]. Another visualization option is the creation of biophysical indices on the fly to enhance certain aspects of the water [76]. This section is implemented so that incorporating new biophysical indexes is effortless; the user will only have to select the image and indicate the algebraic formula associated with the index and generated on the fly.

**Figure 17:**
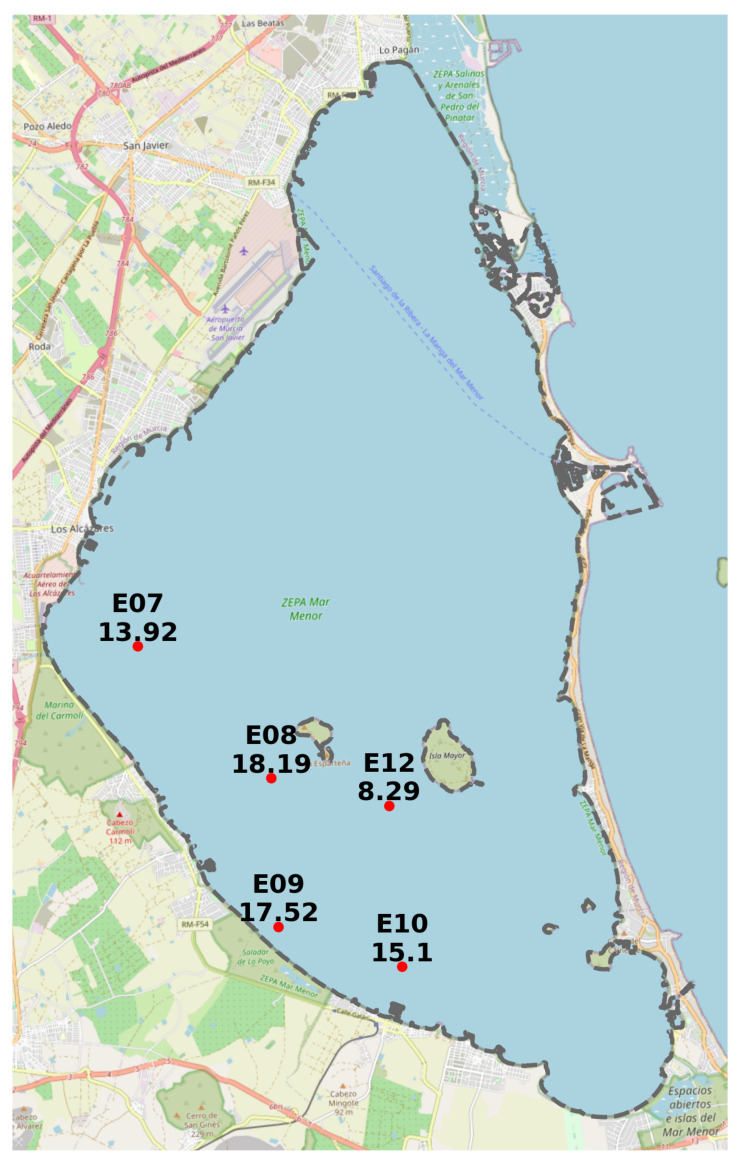
Points with nitrate measurements higher than 6.45 μmol NO${}_{3}$/L, on 16 May 2022.

In the nutrients section, there is access to the combination of the three nutrient datasets (NUTRIMM, NUTRIALB and NUTRICARM). For the entire time series, it is possible to consult the massive data in tabular form, the situation of the levels of each of the measurements concerning Royal Decree 817/2015 [77], which establishes the criteria for monitoring and evaluation of the state of surface waters and environmental quality standards. It also allows the evolution of the measured parameters (ammonium, nitrites, nitrates and phosphates) for the time series, totally or partially. This section, regarding images, allows for generating interpolation maps of the different parameters such as the one represented in Figure 14 or maps in which, at a glance, it can be observed which measurement points present values higher than those established in Royal Decree 817/2015. Figure 17 shows an example of this functionality; it shows five points that, on 16 May 2022, presented nitrate values higher than those established in the Royal Decree (6.45 μmol NO${}_{3}$/L).

**Figure 18:**
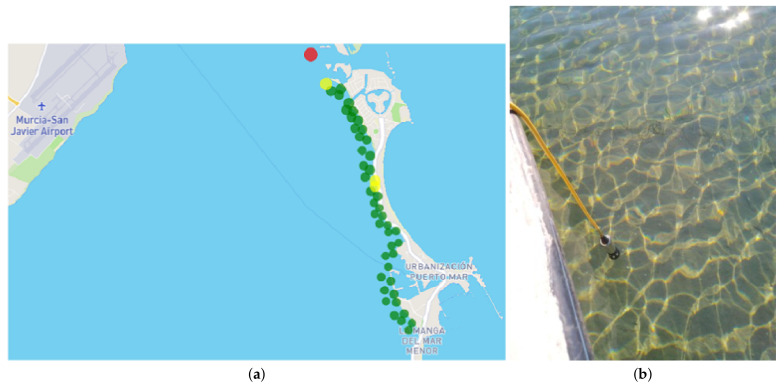
Digital oximeter measurement campaign of 13 July 2022. (**a**) map with the locations of the measurements; (**b**) photograph of the state of the water at a specific measurement point.

Finally, all the data generated in the particular digital oximeter measurement campaigns can be consulted. This section can consult the data collected in the field sheet with the measurements, the photographs taken during the measurements or a situation map with the locations of the measurements configured with a representative symbology of the value obtained. In this map, the green color represents oxygen values above 4 mg/L, the yellow values between 2 and 4 and the red color values below 2. Figure 18 shows the map provided with the measurements of 13 July 2022, together with a photo of one of the points.

##### Campo de Cartagena Monitoring Zone

Within the Campo de Cartagena monitoring area, we find the following options: Plots Monitoring, Meteorology, Basin Monitoring and Hydrology.

The Plots Monitoring option bases all its functionality on the SATELITE dataset; it offers options to perform individualized monitoring of the existing plots in the selected monitoring area. This option provides a query for a specific plot where one can see information regarding the location of the plot, and the NDVI statistics for the whole time series of the selected crop season, both in a table and graphical format. The number of pixels clustered by value intervals (Pixel Clusterer task in Section 5.2.2), information regarding the number of detected crop cycles, and a mosaic of the selected plot with the Sentinel-2 images of the whole time series to visually corroborate the crop cycles.

**Figure 19:**
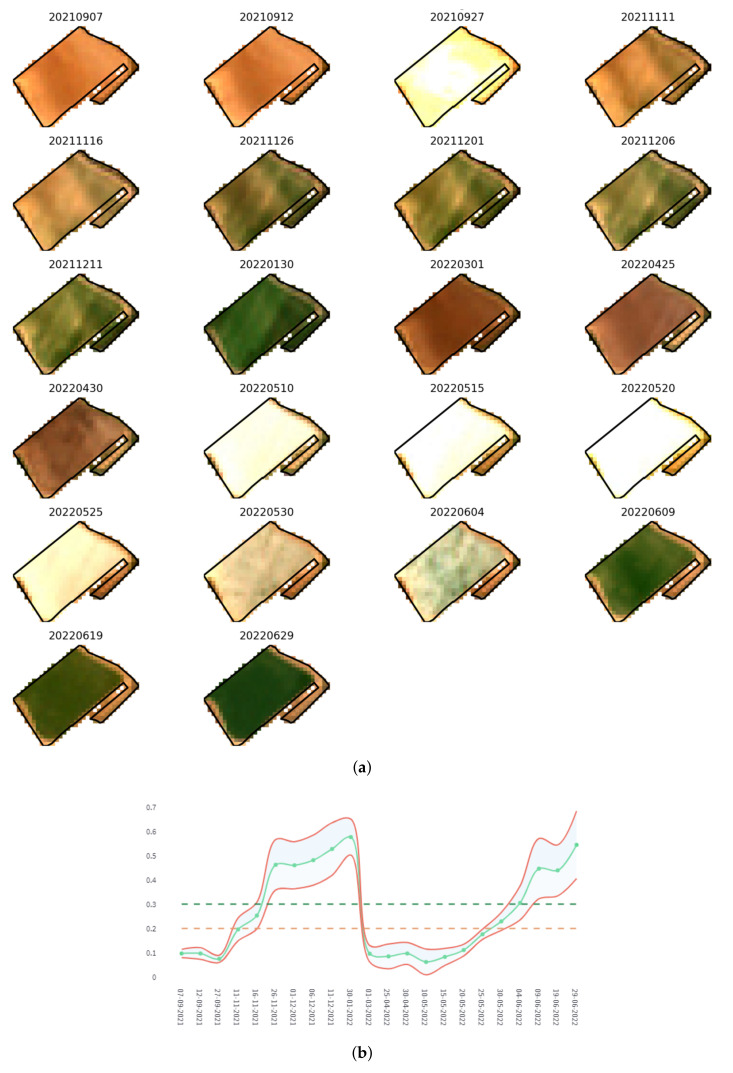
Sentinel-2 and NDVI monitoring of a plot in the Campo de Cartagena for the 2022 time series. (**a**) Sentinel-2 mosaic with the status of a plot for the entire time series of the 2022 campaign; (**b**) graph generated with the NDVI of the 2022 time series for one plot.

Figure 19 shows an example of monitoring a specific plot in the Campo de Cartagena for the 2022 campaign. It shows a mosaic of Sentinel-2 images of the complete time series for the selected plot (Figure 19a) and a graph representing the NDVI index [78], together with a margin of error (±σ × 2), and the delimiting marks of cultivated soil (green dashed line) and bare soil (brown dashed line) (Figure 19b). The dates included in the time series are from 1 September 2021, to 31 August 2022. Two crop cycles can be intuited in it, the first between 30 January 2022, 10 May 2022, and the second from 9 June 2022.

The Meteorology option uses as a data source the set labeled SIAM. It allows for consulting the massive data on the 14 stations included in the study area. For a specific station, it offers options to query massive data for the whole time series and its graphical representation for all the measured parameters. Station data can be consulted for a specific date as a table and a graph. In addition, interpolations can be made for a specific date or a range of dates, and a zonal statistic can be obtained to know the accumulated precipitation in the areas of Decree-Law 2/2019 [79] or the sub-basins of the Campo de Cartagena.

The Basin Monitoring option generates monitoring information of the Mar Menor watershed basin both at the level of annual evolution and at a specific date. The Scientific Data Analysis application section relies on the Satellite data set type. A Sentinel-2 based orthophoto with different color compositions of the whole study area is shown at a specific date level. In addition, it shows a relation between the total surface and irrigated surface, both grouped by type of SIGPAC [80] land use. For the annual evolution, through the NDVI index, the evolution of the estimated irrigated area for the selected crop year is provided.

**Figure 20:**
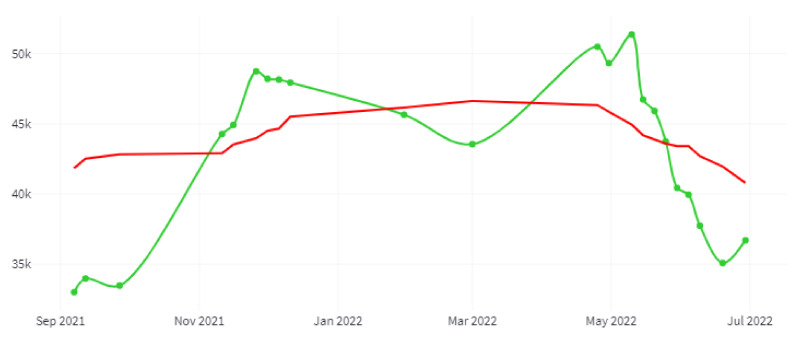
Evolution of irrigated surface area expressed in hectares for the 2022 campaign in the study area.

Figure 20 shows a graph of irrigated hectares’ evolution for the 2022 crop year (September 2021–August 2022) and for the entire study area. The green line indicates the number of hectares, and the red line shows a trend generated with a moving average with a window size of 15 days.

Finally, the Hydrology option provides hydrological information for the Campo de Cartagena area. It relies on the PLUVI, PIEZO and CAUDA datasets. This function displays the data of these datasets in the same way. On the one hand, for a specific date, it shows the data of all measurement elements in the form of a map. On the other hand, a time series shows the evolution of the different parameters for a specific point.

#### 5.4.2. Decision Support System and Web Data

Decision-making is based on experience and knowledge from various data sources and specialized technicians’ recommendations. Therefore, a decision support system will convert all the disparate data collected into information to make the best decisions.

**Figure 21:**
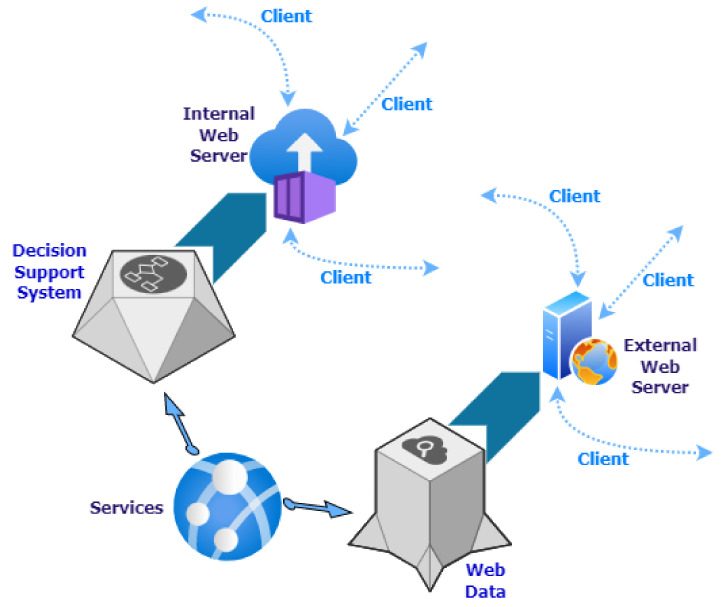
Diagram showing the operation of the Decision Support System and Web Data applications.

This section will discuss the remaining two applications. First of all, Figure 21 shows the schematic diagram with the general operation of both applications.

The Decision Support System application is a continuation of the previous one from the point that the functionalities are first implemented and tested in Scientific Data Analysis. They are later incorporated in this one if they offer what is expected of them. Thus far, it incorporates most of the functionalities of Scientific Data Analysis, although with certain limitations so as not to include elements that could divert it from its primary purpose. Its objective is to help the manager know Mar Menor’s ecological situation and to give clues about the possible causes so that it can make the most appropriate and correct decisions. It offers the data delimited in time, generally to the current month or year. The representation of this data is mostly in graphical and interpolated image format. Data are also included in tabular form, although in a summarized and grouped form. The parameterization options offered are limited. The application is implemented with the JavaScript programming language and its execution environment NodeJS. The application is deployed on an internal server, which is not accessible outside the organization.

The Web Data application is the last to reach the functionalities in the application chain. The functionalities arrive in a significantly reduced form, with fully consolidated and validated data and without offering any parameterization option. Its objective is to offer citizens consolidated information on the state of the Mar Menor in a simple way. To achieve this, it offers a minimal set of information in the form of a graphic or image with interpolation. It is implemented with the JavaScript programming language and its execution environment NodeJS. The application is in the testing phase; when it is finished, it will be deployed on an external server so it can be accessed from the Internet.

## 6. Evaluation and Results

This section describes validating the integrated management platform for the Mar Menor and the Campo de Cartagena Basin from two different perspectives. The first one is strictly focused on the infrastructure and architecture proposed as a solution. Furthermore, the second one is from the point of view of using the platform to improve decision-making.

### 6.1. Infrastructure and Platform Evaluation

One of the critical aspects to be ensured when proposing the platform from a theoretical point of view was flexibility and scalability. Since the beginning of the project, new data sets and functionalities have been incorporated. Initially, only the information related to the manual measurement campaigns carried out with the CTD was available, and then new data such as satellite images or information on hydrology, among others, were gradually added. These additions of new datasets and associated functionalities were made transparently to the existing components without hurting them; this was achieved, on the one hand, thanks to the infrastructure initially proposed and the development methodology employed.

The methodology chosen to achieve this goal was Test Driven Development (TDD) [81]. TDD is based on translating the software requirements into test cases to write their tests, performing the implementation necessary for these tests to pass, and refactoring the written code. This process ensures that everything conforms to the initially defined use cases before the final deployment. In addition to unit tests, integration tests are performed to ensure that the different services work in harmony when working together.

In addition to evaluating the proposed solution through flexibility and scalability, it is evaluated through the response times it offers. For this purpose, a timer is integrated into the unit tests. Table 5 shows the response times based on the type of requests shown in Figure 12. The times were obtained in a controlled environment with the equipment described in Section 4.

The following is a detailed description of the data represented in the table above and how they were obtained:

- Vector Information: vector type information that can be given in point or polygon form. It is represented on a base map offered by third parties (Cartodb or Openstreetmap), which may cause a delay in the request. The minimum time is obtained by retrieving the 21 measurement points in the Mar Menor water body from the CTD dataset, and the maximum time comes from retrieving a polygon corresponding to a crop plot;
- Interpolation: image-type information in base64 format, georeferenced and with a spatial resolution of 20 m for the Mar Menor and 100 for the Campo de Cartagena. It is generated through an interpolation method (IDW, Kriging, Spline) that estimates cell values from real data. The minimum time is obtained when generating the interpolation of nutrients over the Mar Menor, and the maximum time corresponds to interpolating the agrometeorological data of the study area;
- Raster on the fly: image in base64 format, georeferenced with a spatial resolution of 20 m for the Mar Menor and 100 for the Campo de Cartagena. It represents a satellite image as a combination of bands of the visible spectrum or as the result of an algebraic operation between the different bands of a satellite image. The shortest response time is obtained by representing the true color image of the Mar Menor, as shown in Figure 5. The longest response time corresponds to the generation of the NDVI index of the entire Campo de Cartagena;
- Simple Query: retrieval of alphanumeric information involving a maximum of 1 table with less than 100 records between them. The minimum response time is obtained by retrieving the flow data for a specific date. The maximum response time is obtained when querying nutrient data on the Mar Menor for a specific date;
- Complex Query: retrieval of alphanumeric and geospatial information involving at least two tables. It may include aggregation operations. The minimum response time is obtained by retrieving the piezometric data and the locations for a specific date. The maximum response time is obtained by querying the raw data retrieved by the multiparameter oceanographic multisonde for a given day;
- Query with post-processing: retrieval of alphanumeric and geospatial information without table and record limits. It involves post-processing of the initially retrieved data. The minimum response time is obtained with the geometric mean generation by water volume with the oceanographic sonde data. The maximum response time is obtained by generating the table with the ratio of hectares per value range grouped by SIGPAC use from the NDVI index;
- Graph with one series: graphical representation of a single series. It includes the data recovery time. The minimum time corresponds to the representation of the transparency values for the whole historical series. The maximum is obtained by generating the representation of the values retrieved by the oceanographic sounder for a specific parameter and date;
- Graph with multiple series: graphical representation with multiple series. It includes the time of data collection. The minimum time corresponds to the representation of the agrometeorological data of a parameter and a specific station for the whole historical series. The maximum is obtained by generating the representation of the values retrieved by the oceanographic sonde for the parameters of a specific date;
- Graph with multiple series and scales: graphical representation with multiple series and each one with a different scale. It includes the time of data collection. The minimum time corresponds to the representation of the agrometeorological data of four different parameters for a specific station and the whole historical series. The maximum is obtained by generating the representation of the global values of several parameters of the oceanographic sounding for the whole time series;
- Prediction by downloading file: development of a prediction through the Machine Learning model after downloading the serialized model; the download is performed on the client, so bandwidth is a determining factor;
- Prediction with downloaded file: launching a prediction through the Machine Learning model, with the file available in the client.

### 6.2. Use of the Platform as an Improvement in Decision-Making

The proposed platform brings together the necessary knowledge base to improve decision-making. It is presented to the user through a user-friendly computer system to develop complete and interactive decision support for the follow-up and monitoring of the Mar Menor and its watershed basin. The platform will allow managers to make the best decisions to improve the ecological conditions of the lagoon and simultaneously reduce the emission of components that affect it.

Using the experiences of the accumulated data and the information provided by the platform, it is possible to predict the evolution of the lagoon’s condition and prevent future environmental problems. Consequently, it is possible to establish alternatives in monitoring the different elements that affect the lagoon’s state and improve the ecosystem’s maintenance to develop an adequate balance between socio-economic results, environmental values and agricultural yield.

At the level of practical application and support for decision-making on the monitoring area of the Mar Menor and through the CTD data, the proposed solution allows knowing the ecological status with a high level of detail. For example, it is possible to detect an episode of oxygen deficit even at depth level. Consult the biophysical evolution of a given measurement point for the entire historical series or obtain the general state of the lagoon for a specific date or for the entire time series. This section is beneficial in detecting periods of hypoxia and determining the extent of the affected zone. Concerning nutrients, it offers information to analyze the evolution of the different types for a specific date or for the entire time series, as well as to determine problems of high nutrients and to delimit the affected zone. In addition, high-resolution satellite images offer the possibility to visualize the state of the lagoon with simple view or to enhance biophysical parameters, such as chlorophyll or turbidity, through the generation of indices.

**Figure 22:**
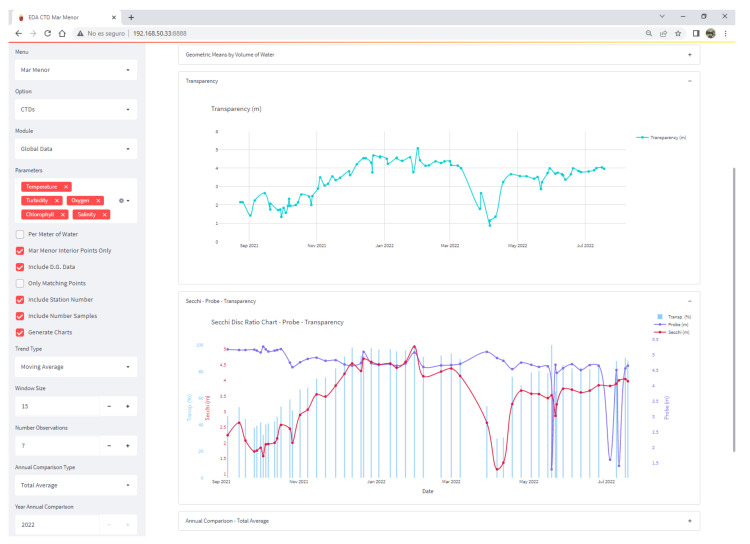
Example of the screen of the Scientific Data Analysis application with the global data of the Mar Menor.

Figure 22 shows one of the screens of the Scientific Data Analysis application, specifically the one that reports the general state of the Mar Menor. The left side shows the large number of parameterization options it offers; in the central part, graphs of transparency are displayed, together with their relationship with the measurements made with the Secchi Disk and by the oceanographic sonde.

As examples of practical applications to aid decision-making in the Campo de Cartagena monitoring area, agrometeorological and hydrological data are used to calculate the amount of precipitated water in the basin and to monitor the runoff produced in the watershed and the amount of water entering the Mar Menor during extreme events. It also allows visualization of the piezometric levels and nitrate content of the Quaternary aquifer. On the other hand, thanks to satellite images and geographic information systems, it is possible to monitor at the crop level. Among the options offered, we can obtain general information for the entire watershed or information at the agricultural plot level. It allows monitoring land use through vegetation indices, detecting the number of crop cycles of each plot or obtaining the number of irrigated hectares and their evolution for an agricultural campaign.

## 7. Conclusions

Using data science supported by satellite images allows us to obtain information on the eutrophication processes of coastal lagoons, as in the case of the Mar Menor. In addition, they play a crucial role in vegetation monitoring as they show valuable information to study the state of vegetation cover in the watershed through vegetation indices and biophysical indices of water quality. These technologies provide potent tools for monitoring, protecting and restoring vulnerable ecosystems such as the Mar Menor.

This work arises from the need to bring together all the variables that condition the ecological status of the Mar Menor and its environment and provide different information according to user profiles. At the regional level, we consider it the first system capable of generating information on the lagoon and its environment from multiple data sources to improve decision-making. The existing data come from different sources and in different formats. Finally, a solution has been developed based on flexibility, scalability and sustainability in terms of infrastructure. At all times, free software and open source products create components with a low level of coupling and very high independence that allows the incorporation of new components transparently to the rest. In this work, it is possible to integrate the massive amount of input data into a single data source and process them appropriately to transform them into useful information for different user profiles. It has been a priority to provide a geospatial component to these data to locate them on a map and thus better pinpoint the lagoon area most affected by specific special episodes or anomalies. The user profile that can use the system ranges from users with a scientific or data analyst profile to ordinary citizens, including users with managerial responsibilities in decision-making. The information generated can be displayed in different ways, from a table of raw data to graphics of different complexity, images with a geospatial component, maps with alphanumeric information, interpolations or raster, and with different levels of parameterization. In addition, it has been possible to unify more than 12 million records and work together with all of them regardless of their origin or format; the volume of data has grown exponentially in the last year, but they have been able to be manipulated without problems thanks to the flexible and decoupled infrastructure designed.

Generally, good response times are observed regarding the platform’s usability. However, response times for specific types of resources can be striking. The types of resources affected are those related to raster geospatial information and complex queries with post-processing. Although the operations carried out concerning raster are of high complexity, the casuistry could be analyzed in depth to propose an approach that would reduce these response times, for example, by creating a physical caching system on the server. About queries with post-processing, high response times occur when working with massive volumes of data. As in the previous case, it would be necessary to analyze each case and approach the problem from a different perspective; for example, the data sets could be divided by annuities to work with smaller data sets.

Many data sets have been integrated with sufficient coherence to establish causal relationships between the functionalities offered for advanced data analysis and decision support. The options offered are high and with a high level of parameterization. The developed solution makes it possible to anticipate extraordinary episodes that may occur in the Mar Menor, even being able to delimit the area affected. New functionalities to be developed include the refinement of the relationships between the different parameters to know the relationship between, for example, the relationship between nutrients and the rise in temperature and its direct impact on the saline coastal lagoon [82].

Among the future ways to be implemented is incorporating new data sources such as the data generated by automatic buoys whose installation is in the testing phase or the future nutrient auto-analyzers installed shortly in the lagoon. In addition, it would be interesting to incorporate data from the network of stations of the State Meteorological Agency (AEMET), which covers the study area of this work, in order to complete the existing data series. Another line to follow is to work with greater integration between the input data sets to generate a warning and forecasting system. Finally, we intend to go deeper into automatic and deep learning to generate a model capable of detecting anomalies in the water mass, such as pockets of anoxia or fluctuations in chlorophyll or turbidity, using an image in raster format.

## Figures and Tables

**Table 1:** Data used by the proposed platform.

Code	Origin	Parameters	Type	Points	Zone	Period	Periodicity
CTD	CARM	7	Biophysical	21	Mar Menor	2021–2022	weekly
SIAM	IMIDA	5	Meteorological	14	C.Cartagena	2016–2022	daily
NUTRIMM	IMIDA	4	Nutrients	21	Mar Menor	2021–2022	weekly
NUTRIALB	IMIDA	4	Nutrients	15	Mar Menor	2021–2022	weekly
NUTRICARM	CARM	4	Nutrients	14	Mar Menor	2021–2022	weekly
PIEZO	SAIH	5	Hydrology	20	C.Cartagena	2021–2022	daily
CAUDA	SAIH	1	Hydrology	8	C.Cartagena	2021–2022	daily
PLUVI	SAIH	1	Hydrology	27	C.Cartagena	2021–2022	daily
SENTINEL	ESA	11	Satellite	130	Study area	2018–2022	weekly
LANDSAT	NASA	11	Satellite	48	Study area	2021–2022	weekly

**Table 2:** Volume of data by typology.

Data Type	Volume (Records)
Biophysical	349,000
Meteorological	36,000
Nutrients	10,000
Hydrology	32,000
Satellite	11,800,000
Total	12,227,000

**Table 3:** Description of the sensors that make up the SIAM stations.

Sensor	Brand	Model
Thermo Hygrometer	Vaisala	HMP45AC
Radiometer	Kipp & Zonen	CMP6
Pluviometer	Young	52203
Anemometer	Young	05103-5

**Table 4:** Weighting factors for the geometric mean by water volume.

Isobata	Area (km${}^{2}$)	Vol (hm${}^{3}$)	Vol Acu (hm${}^{3}$)	W (%)
0 m–1 m	135.20	130,197	657,488	19.80
1 m–2 m	126.34	122,825	527,291	18.68
2 m–3 m	119.43	116,049	404,467	17.65
3 m–4 m	112.53	107,870	288,417	16.41
4 m–5 m	102.56	94,580	180,547	14.39
5 m–6 m	84.78	68,092	85,967	10.36
6 m–7 m	45.50	17,871	17,875	2.72
7 m–8 m	0.39	0.0004	0.0004	0.00

**Table 5:** Response times (ms) by request type.

Resource Type	Response Times (ms)		
	Minimum	Medium	Maximum
**Geospatial Data**			
*Vectorial Information*	10	67	164
*Interpolation*	1181	2221	3996
*Raster on the fly*	1430	4752	11,020
**Data Table**			
*Simple Query*	3	43	95
*Complex Query*	120	261	384
*Consultation with post-processing*	503	2898	8419
**Plot Data**			
*Chart with a series*	29	58	91
*Chart with multiple series*	176	319	572
*Chart with multiple series and scales*	205	343	622
**ML Model**			
*Prediction by downloading file*	-	2258	-
*Prediction with downloaded file*	-	246	-

## Data Availability

Data sharing not applicable.

## Appendix A. Selected Literature Criteria

The selected literature has been chosen according to the following criteria:

- The journal languages used are English and Spanish.
- The articles with the most significant impact have been selected on a similar topic.
- The most recent articles have been selected in articles with a similar impact.
- The databases used to obtain articles were Google Scholar, Mendeley and ResearchGate.
- The Google search engine is used to search for non-scientific information, such as governmental websites, libraries on which the proposed solution is based, or data sources used in this work.
- The content of the journals corresponds to coastal lagoons, watershed basins, environment, water quality, remote sensing and agriculture.
